# The gastric microbiota in patients with Crohn’s disease; a preliminary study

**DOI:** 10.1038/s41598-021-97261-z

**Published:** 2021-09-09

**Authors:** Jerzy Ostrowski, Maria Kulecka, Iwona Zawada, Natalia Żeber-Lubecka, Agnieszka Paziewska, Katarzyna Graca-Pakulska, Krzysztof Dąbkowski, Karolina Skubisz, Patrycja Cybula, Filip Ambrożkiewicz, Elżbieta Urasińska, Michał Mikula, Teresa Starzyńska

**Affiliations:** 1 Department of Genetics, Maria Skłodowska-Curie National Research Institute of Oncology, Roentgena 5, 02-781 Warsaw, Poland; 2 Department of Gastroenterology, Hepatology and Clinical Oncology, Centre of Postgraduate Medical Education, Warsaw, Poland; 3 Department of Gastroenterology, Pomeranian Medical University in Szczecin, Unii Lubelskiej 1, 71-252 Szczecin, Poland; 4 Department of Pathology, Pomeranian Medical University in Szczecin, Szczecin, Poland

**Keywords:** Crohn's disease, Bacterial genetics

## Abstract

The gastric microbiota in Crohn’s disease (CD) has not been studied. The purpose of the study was to evaluate differences of stomach microbiota between CD patients and controls. DNA was extracted from gastric mucosal and fluid samples, from 24 CD patients and 19 controls. 16S rRNA gene sequencing identified 1511 operational taxonomic units (OTUs), of which 239 passed the low abundance and low variance filters. All but one CD patients were HP negative. Fifteen bacterial phyla were identified in at least one mucosal or fluid site. Of these, *Bacteroidota* and *Firmicutes* accounted for 70% of all phyla. *Proteobacteria*, *Actinobacteriota*, and *Fusobacteriota* combined accounted for 27%. There was significant difference in the relative abundance of *Bacteroidota*, *Proteobacteria*, *Fusobacteriota*, and *Campilobacterota* between CD patients and controls only in gastric corpus samples. In gastric liquid, there was a significant difference only in *Actinobacteriota*. Pairwise comparison identified 67 differentially abundant OTUs in at least one site. Of these, 13 were present in more than one comparison, and four differentiating OTUs (*Neisseriaceae*, *Neisseria*, *Absconditabacteriales*, and *Microbacteriaceae*) were identified at all tested sites. The results reveal significant changes in gastric microbial profiles (beta diversity, phylum, and individual taxa levels) between *H. pylori-*negative CD patients and controls.

## Introduction

Crohn's disease (CD) is a chronic, relapsing inflammatory condition of the gastrointestinal tract (GIT) that affects millions of people (mostly young) worldwide; the condition can have life-threatening complications^1^. CD can affect any part of GIT, from the mouth to the anus. Prospective endoscopic testing of 1015 adult CD patients identified macroscopic and microscopic findings within the upper GIT of 11.7% of patients; mostly, these findings were unspecific and of uncertain clinical relevance, with concomitant involvement of the ileocolonic region in 60% of cases^2^. However, as reviewed recently^3^, macroscopic changes in the upper GIT are found in 50–70% of patients with CD.

While hydrochloric acid and proteolytic enzymes present in gastric fluid provide a protective barrier against ingested bacterial pathogens, the stomach is not a sterile organ. The gastric microbiome comprises Gram-negative and Gram-positive commensal bacteria, which reside in both the gastric mucosa and gastric fluid^4^. *Proteobacteria* is the predominant phylum in *Helicobacter pylori* (HP)-infected subjects, comprising 72–99% of all bacteria in the gastric mucosa^5^. HP, which colonizes the gastric epithelium, infects half of the world’s population, with higher prevalence observed in developing countries. HP infection is associated with chronic gastritis, peptic ulcer disease, gastric cancer, and mucosa-associated lymphoid tissue lymphoma. Regardless of HP status, other phyla, including *Actinobacteria*, *Bacteroidetes*, *Firmicutes*, and *Fusobacteria*, are also detected consistently^5,6^.

The prevalence of HP infection in patients with inflammatory bowel disease (IBD) is low, and both case–control and ecological studies clearly show an inverse correlation between the prevalence of HP infection and development of CD^7^. Furthermore, HP infection is associated independently with lower incidence of fistulizing/stricturing disease and/or less active colitis in CD patients^8^. Thus, HP may have a direct immunomodulatory effect on immunopathological processes that protect against CD development, or it may be a marker of protective effects resulting from exposure to other infections in early life; therefore, there may be a relationship between gastrointestinal dysbiosis and CD-related alterations in the immune response.

The microbial density in the stomach ranges from 10^2^ and 10^4^ CFU/g, although this fluctuates considerably depending on the pH of the gastric lumen^6^. While host genetic background has little effect in shaping the gastric microbiota^9^, it is controversial whether there might be ethnic and geographic differences in the diversity of the gastric microbiome^6^. The presence of a gastric microbiota can be documented by both conventional methods and culture-independent molecular techniques.

CD-related gastric dysbiosis has not been studied. Here, we used 16S rRNA gene amplicon sequencing to identify meaningful differences in the gastric mucosal and gastric fluid microbiomes of HP-negative CD patients and HP-negative controls.

## Materials and methods

### Patients

From January 2019 to July 2019, we prospectively recruited 24 consecutive CD patients [11 women and 13 men; median age, 34.5 years (range 19–64 years)] with available clinical information (Table 1). CD was diagnosed by experienced gastroenterologists during a standard diagnostic work-up using the Porto criteria, modified in accordance with ECCO guidelines^10^. Patients were recruited during a course of hospital treatment or during a scheduled visit to the out-patient clinic at the Department of Gastroenterology, Pomeranian Medical University in Szczecin. According to the CD activity index, the CD patients were in disease remission or had disease with mild clinical activity. Seventeen of twenty-four patients (70.8%) had ileocolic disease. The control group comprised 19 patients [15 women and four men; median age, 38 years (range 22–72 years)] who underwent gastroscopy as a routine diagnostic procedure for dyspepsia (12 subjects) or gastric cancer surveillance (seven subjects). All enrolled CD patients and controls were Polish Caucasians.

**Table 1:** Clinical characteristics of enrolled patients with Crohn’s disease.

Variable	Data
**Disease extension**	
Ileal	7 (29.2%)
Ileocolonic	17 (70.8%)
Fistulising disease	6 (25.0%)
Stricturing disease	13 (54.2%)
**Therapy at the time of enrollment**	22 (91.7%)
Azathioprine	11 (45.8%)
Steroids	9 (37.5%)
Biologic	2 (8.3%)

None of the study participants received antibiotics or proton pump inhibitors within the 6 months prior to enrollment, and none had undergone gastric surgery/endoscopic treatment for gastric lesions or *H. pylori* eradication therapy.

### Gastric biopsy specimens and juice collection

Following fasting (≥ 10 h), subjects underwent routine upper gastrointestinal endoscopy, and samples of gastric mucosa and gastric fluid were collected. The biopsy collections comprised 7 specimens: of these, gastric biopsy sample from each antrum and corpus site were sent for histopathological examination, two from each site were used for microbiome assessment, and one from the antrum was used for a rapid urease test. Gastric liquid was collected by suctioning gastric juice through a sterile tube inserted into the canal of an endoscope.

The gastric biopsy specimens used for histological examination were fixed in buffered 10% formalin and embedded in paraffin and kept at room temperature. Serial sections were cut and stained with hematoxylin and eosin, and then examined by an experienced pathologist. Histologic evaluation was based on the updated Sydney system. Assessed histological parameters included chronic inflammation, histological activity of inflammation, glandular atrophy, intestinal metaplasia and the presence of granulomas.

The gastric biopsy specimens and gastric fluid samples used for 16 s rRNA sequencing were kept at − 80 °C and then transported to the Department of Genetics on dry ice.

### DNA extraction and 16S rRNA sequencing

Genomic DNA from gastric biopsy specimens and juice samples was extracted and purified using the QIAamp DNA Mini Kit (QIAGEN, Germany). DNA concentrations were measured using a Nanodrop ND-1000 spectrophotometer. 16S rRNA gene libraries were sequenced on an Ion Torrent Personal Genome Machine (PGM) platform (Thermo Fisher Scientific, USA) using Ion PGM™ Hi-Q™ View OT2 and Ion PGM™ Hi-Q™ View Sequencing Kits. Bacterial 16S rRNA libraries were prepared using an Ion 16S™ Metagenomics Kit (which allows a consensus view across six regions: V2, V3, V4, V6–7, V8, and V9) and an Ion Plus Fragment Library Kit, as previously described^11^.

The sequenced data were deposited on the PRJEB43132.

### Data and statistical analysis

For 16S rRNA analysis, unmapped BAM files were converted to FASTQ using Picard’s^12^ SamToFastq. Additional steps of the analysis were performed using Mothur software^13^ version 1.38. FASTQ files were converted to the FASTA format. Only sequences that were 200–300 bases in length, with an average base quality of 20 in a sliding window of 50 bases, and a maximum homopolymer length of 10, were included. Chimeric sequences were identified by the UCHIME^14^ algorithm using default parameters, with internal sequence collection as the reference database. Chimeric sequences were removed and the remaining 16S rRNA sequences were classified using the Wang method and the SILVA^15^ bacterial 16S rRNA database for reference (release 138); the bootstrap cut-off was 80%.

PCoA (principal coordinates analysis) of all samples (including HP-positive samples) was performed using MEGAN software version 5.7^16^, with the Bray–Curtis index as a distance measure. Further analysis (excluding HP-positive samples) was performed with R package MicrobiomeAnalystR, version 0.0.0.9000^17^. Taxa with low prevalence (< 5 counts and present in < 10% of samples) and variance (20% and 30% for LDA discriminant analysis of operational taxonomic units (OTUs) with the smallest IQR values) were filtered out. Alpha diversity analysis was performed using the Shannon index as an indicator. The Mann–Whitney U-test used to assess the statistical significance of differences between healthy controls and CD patients. PCoA was performed using the Bray–Curtis index as a distance measure. PERMANOVA was used to test the significance of clustering patterns. Differential abundance of taxa was assessed using the metagenomeSeq method^18^ and LDA discriminant analysis, based on a method from LEfSe software.

### Ethical considerations

The study was performed in accordance with the ethical standards of the local bioethical committee and in accordance with the principles of the 1964 Declaration of Helsinki. All subjects provided written informed consent prior to participation. The study was approved by the local ethics committee (Bioethics Committee of the Pomeranian Medical University in Szczecin: KB-0012/17/19, 2019/dated 14.01.2019).

## Results

### Study groups

Gastric mucosa biopsy and gastric juice samples were collected from 43 subjects; 24 CD patients and 19 patients undergoing routine diagnosis of dyspeptic symptoms or healthy family members enrolled in the gastric cancer surveillance program. Among CD patients, in all but three (21/24, 87.5%) different abnormalities were found in upper endoscopy. Inflammation of gastric mucosa, a bamboo joint-like appearance (BJA) in proximal part of gastric body or/and fundus, and antral erosions were the most common stomach lesions, demonstrated in 16, 12 and 7 patients, respectively. One patient had multiple gastric ulcers, deformation and stenosis of antral part. Ten CD patients developed changes in duodenum, including erosions/mucosal redness-edema found in 6 patients, ulcers found in 3 patients, and stenosis in one patient.

In all HP-positive subjects histologic evaluation showed chronic active gastritis (mild, moderate, or marked).

Among 22 HP-negative CD patients (microscopic findings were unavailable for one CD patient), all patients had chronic gastritis (mostly inactive—19 patients, usually mild).

In most HP-negative control subjects (9 out of 13) microscopic evaluation revealed normal gastric mucosa .

### 16s rRNA sequencing data

**Figure 1:**
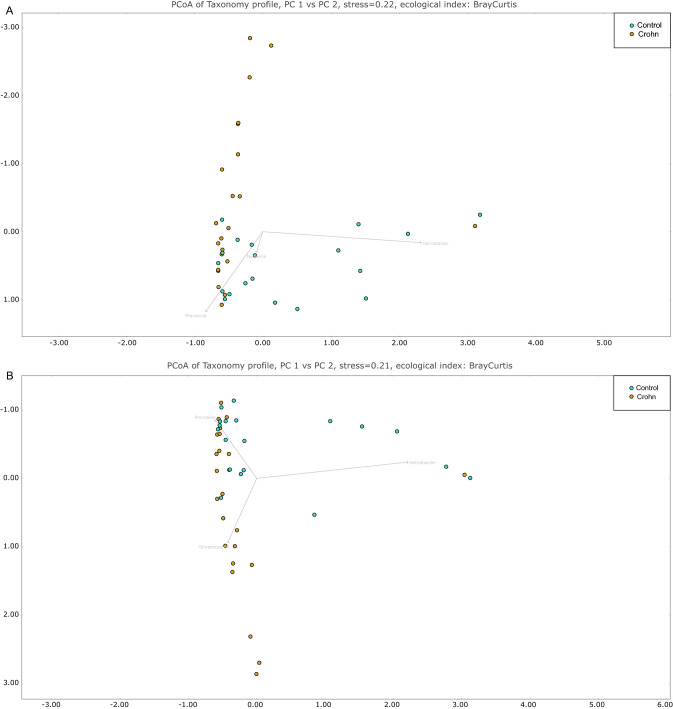
Principal coordinates analysis of all studied samples using the Bray–Curtis index as a distance measure. (**A**) Corpus; (**B**) antrum.

Bacterial DNA extracted and purified from gastric biopsy specimens and gastric liquid samples was used for PCR amplification of bacterial 16S hyper-variable regions. Prepared libraries were sequenced using the PGM platform. For each sample, 5632–246,799 (median 103,271; mean 109,467) of the generated reads passed quality control. Overall, 62–100% of sequences were classified using SILVA database version 138 as a reference and were assigned to Bacteria and Archaea taxa. PCoA performed using taxa data from mucosal samples (based on Bray–Curtis distances) revealed two distinct clusters that differentiated HP-positive from HP-negative samples (Fig. 1A,B). One out of twenty-four (4.2%) CD patients and 6 out of 19 (31.6%) control subjects were infected with HP; the sequencing data confirmed the positive results of a rapid urease test in all cases. In HP-infected individuals, the relative abundance of *Helicobacter* genus was significantly higher in gastric mucosal samples (median = 56%; range 20–98%) than in gastric fluid samples (median = 2; range 1–13%).

**Figure 2:**
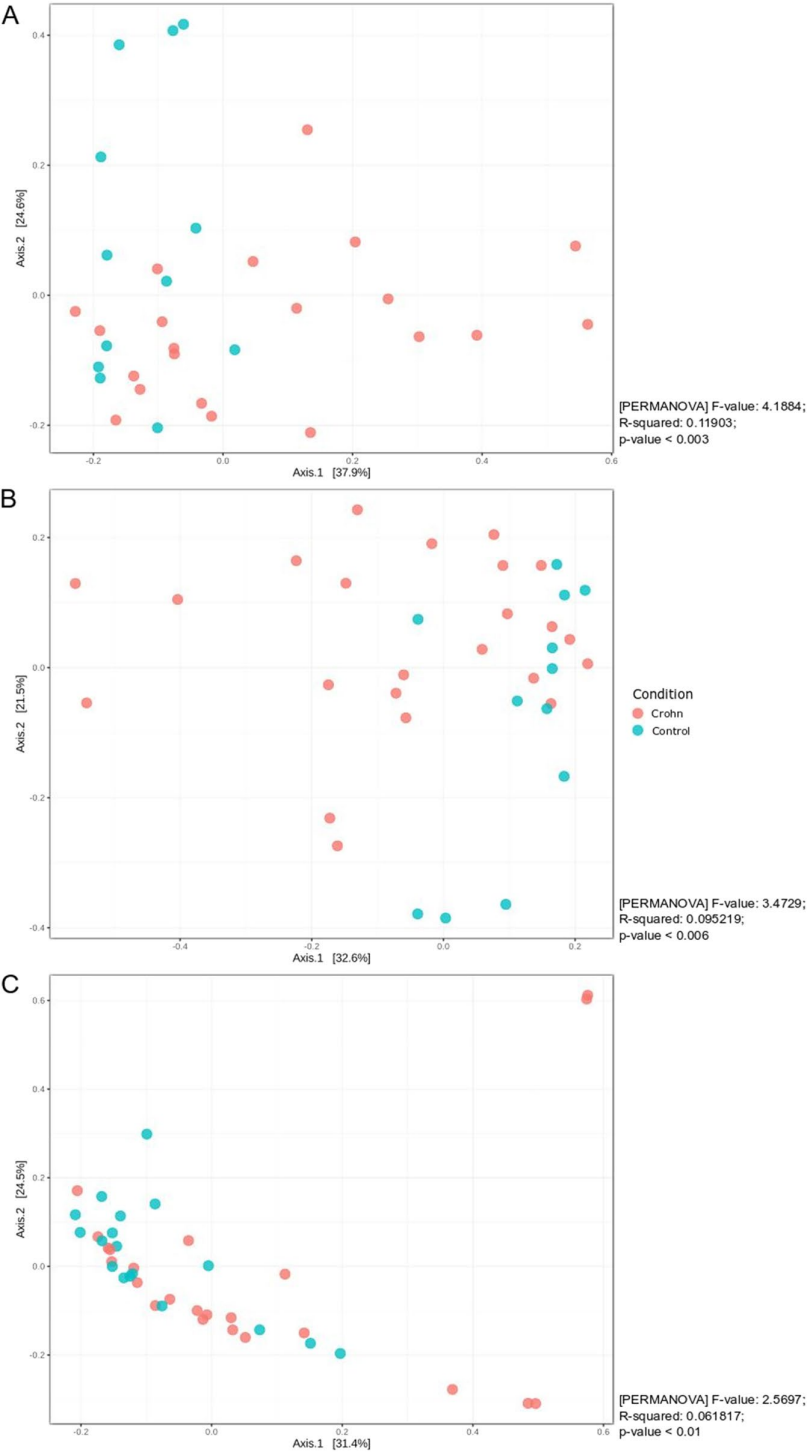
Principal coordinates analysis of HP-negative samples using the Bray–Curtis index as a distance measure. The significance of the clustering patterns was tested using PERMANOVA. (**A**) Corpus; (**B**) antrum; (**C**) gastric fluid.

After excluding HP-positive samples, there was no statistically significant difference in alpha diversity (measured using Shannon’s index) between samples collected from either test site in CD patients and controls. By contrast, PCoA analysis based on a Bray–Curtis index revealed that clustering patterns in CD patients were significantly different from those in controls at all tested sites (Fig. 2).

### Taxonomic analysis

**Figure 3:**
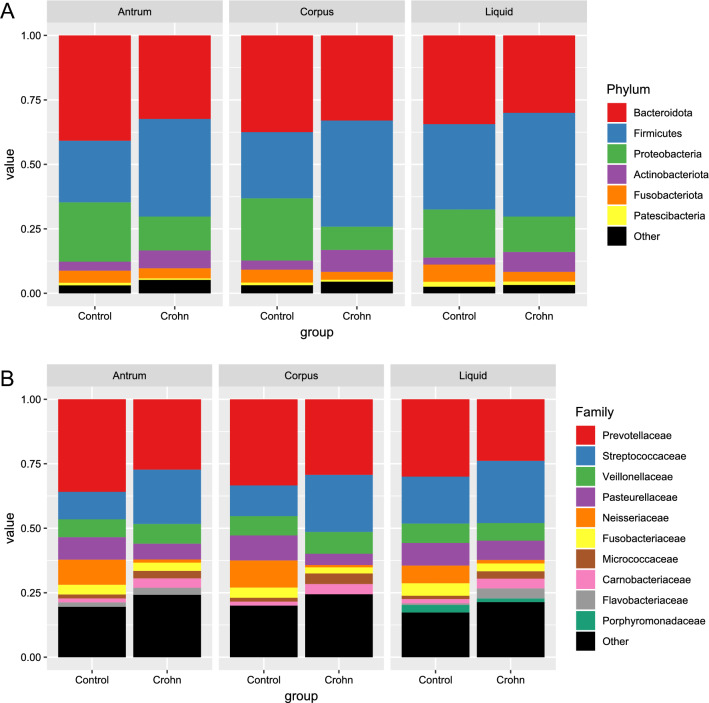
Mean taxa abundances on phylum (**A**) and family (**B**) levels. Only taxa with mean abundance higher than 0.02 (families) or 0.01 (phyla) are included.

Categorization of sequence reads among both mucosal and gastric fluid samples collected from HP-negative controls and CD patients into OTUs identified 1511 OTUs, of which 239 passed the low abundance and low variance filters. When the OTUs were categorized into phylum subgroups, 15 bacterial phyla were identified in at least one gastric mucosal or liquid sample, of which nine, six, and five phyla were found consistently in the corpus, antrum, and gastric fluid, respectively. At the phylum level, *Bacteroidota* and *Firmicutes* were dominant in all gastric sites, with a total abundance of around 70%; three phyla (*Proteobacteria, Actinobacteriota*, and *Fusobacteriota*) accounted for ~ 27% of all OTUs, while five phyla (*Patescibacteria*, *Campilobacterota*, *Eukaryota_unclassified*, *Spirochaetota*, and *Cyanobacteria*) accounted for less than 2% (Table 2, Fig. 3A). Although the relative abundance of *Bacteroidota*, *Proteobacteria*, *Fusobacteriota*, and *Campilobacterota* differed significantly in CD patients and control subjects only in gastric corpus samples, and *Actinobacteriota* differed only in gastric liquid samples, the profiles of mean abundancy changes were very similar at all three gastric sites (Table 2). The *Firmicutes/Bacteroidota* ratio was higher in gastric samples from CD patients than in those from control subjects (median ratio in the corpus, antrum, and gastric liquid = 1.11, 1.05, and 1.30 for controls, and 0.64, 0.57, and 0.84 for CD patients, respectively; P-value = 0.0037, 0.00054, and 0.09, respectively). Thus, differences were statistically significant for both mucosal sites but not for gastric liquid. On family level, representants of 10 families accounted for more than 75% of all reads, with *Prevotellaceae* and *Streptococcaceae* dominating all sites (Fig. 3B).

**Table 2:** The top ten most abundant phyla, and their relative abundance, in all three types of gastric samples.

	Corpus			Antrum			Liquid			Mean
	CD	Controls	FDR	CD	Controls	FDR	CD	Controls	FDR	
	Mean (SD)	Mean (SD)		Mean (SD)	Mean (SD)		Mean (SD)	Mean (SD)		
*Bacteroidota*	32.97% (15.21%)	37.49% (10.71%)	1.27E−02	32.36% (13.49%)	40.82% (12.88%)	4.99E−01	30.01% (14.29%)	34.38% (10.91%)	9.93E−01	34.67%
*Firmicutes*	41.16% (14.10%)	25.66% (10.06%)	7.06E−01	37.85% (13.65%)	23.88% (7.93%)	5.57E−01	40.21% (20.69%)	33.08% (15.48%)	8.36E−01	33.64%
*Proteobacteria*	9.01% (5.81%)	24.13% (18.45%)	1.58E−03	13.16% (11.60%)	23.00% (16.92%)	3.25E−01	13.73% (14.78%)	18.64% (15.35%)	4.26E−01	16.95%
*Actinobacteriota*	8.51% (7.20%)	3.54% (1.48%)	8.62E−01	6.89% (6.98%)	3.50% (1.32%)	7.62E−01	7.70% (8.26%)	2.75% (1.71%)	3.63E−02	5.48%
*Fusobacteriota*	3.09% (2.24%)	5.01% (3.63%)	1.91E−02	3.91% (3.93%)	4.68% (3.23%)	4.99E−01	3.78% (3.00%)	6.67% (5.13%)	3.02E−01	4.52%
*Patescibacteria*	0.71% (0.75%)	0.99% (0.59%)	1.19E−01	0.61% (0.64%)	1.00% (0.68%)	4.99E−01	1.30% (1.35%)	1.89% (1.37%)	8.36E−01	1.08%
*Campilobacterota*	0.57% (0.76%)	1.73% (3.48%)	1.91E−02	0.46% (0.40%)	0.73% (0.82%)	4.99E−01	0.61% (0.91%)	1.10% (1.00%)	8.08E−01	0.87%
*Eukaryota unclassified*	0.30% (0.75%)	0.22% (0.34%)	9.23E−01	0.49% (1.31%)	0.37% (0.56%)	9.12E−01	0.40% (1.41%)	0.22% (0.78%)	8.08E−01	0.33%
*Spirochaetota*	0.16% (0.17%)	0.13% (0.14%)	4.37E−01	0.25% (0.36%)	0.18% (0.27%)	7.62E−01	0.22% (0.24%)	0.39% (0.55%)	8.80E−01	0.22%
*Cyanobacteria*	0.11% (0.23%)	0.05% (0.07%)	8.62E−01	0.13% (0.22%)	0.10% (0.15%)	6.09E−01	0.13% (0.43%)	0.01% (0.02%)	3.02E−01	0.09%

**Figure 4:**
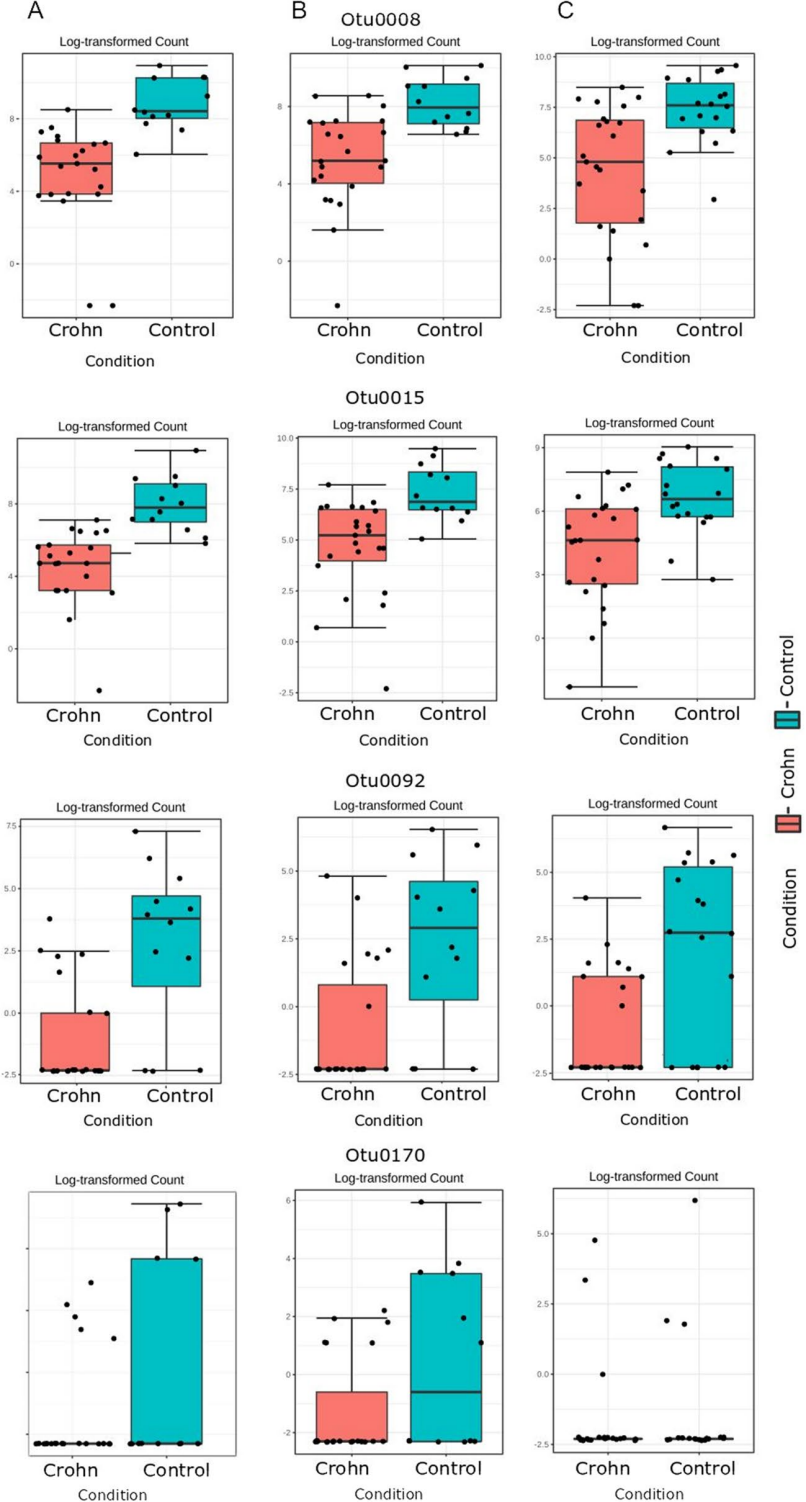
Abundance of taxa that differentiate healthy controls from HP-negative CD patients at all sites, presented as log-transformed counts. (**A**) Corpus; (**B**) antrum; (**C**) gastric liquid.

There were 67 differentially abundant OTUs in at least one site (Supplementary Table 1). Of these, 13 differentiating OTUs were present in more than one comparison (Table 3), and four differentiating OTUs were present in all tested sites (Fig. 4). In general, the largest number of differentially abundant OTUs was found in gastric fluid samples (50 OTUs), whereas 27 OTUs showing differential abundance between CD patients and control subjects were found in the corpus and 7 OTUs—in the antrum. On the other hand, LDA analysis, designed specifically for the biomarker discovery, showed significantly smaller numbers of differential OTUs (Supplementary Table 2, Table 4): 3 each for corpus and antrum and only one for stomach liquid. Most of these taxa are in line with metagenomeSeq results.

**Table 3:** Operational taxonomic units (OTUs) showing differential abundance between *H. pylori*-negative control subjects and CD patients at more than one gastric site in metagenomeSeq analysis.

OTU/taxonomy	FDR-corpus	FDR-antrum	FDR-liquid
Otu0015/*Neisseriaceae*	0.0002	0.01	0.02
Otu0008/*Neisseria*	0.0002	0.005	0.02
Otu0092/*Absconditabacteriales_(SR1)_ge*	0.000413	0.0007	0.0001
Otu0170/*Microbacteriaceae;Candidatus_Aquiluna*	0.002	0.0007	0.02
Otu0191/*Rhodocyclaceae; C39*		0.02	0.01
Otu0103/*Carnobacteriaceae*		0.02	0.05
Otu0012/*Porphyromonas*	0.02		0.05
Otu0062/*Pelomonas*	0.005		0.001
Otu0109/*Ralstonia*	0.04		0.003
Otu0138/*Abiotrophia*	0.02		0.03
Otu0180/*RF39_genus*	0.002		0.00006
Otu0202/*Caulobacter*	0.005		0.02
Otu0183/*Erysipelotrichaceae*	0.047		0.01

**Table 4:** Differential taxa in LDA analysis.

Features	Corpus					Antrum					Liquid				
	Pvalues	FDR	Crohn	Control	LDAscore	Pvalues	FDR	Crohn	Control	LDAscore	Pvalues	FDR	Crohn	Control	LDAscore
Otu0015/Neisseriaceae	1.6E−04	1.6E−02	2.5E+04	3.6E+05	− 5.22	3.4E−04	2.4E−02	3.2E+04	3.3E+05	− 5.17	7.0E−04	6.0E−02	4.0E+04	2.6E+05	− 5.04
Otu0008/Neisseria	6.2E−05	1.3E−02	5.9E+04	6.9E+05	− 5.5	3.4E−04	2.4E−02	8.4E+04	6.4E+05	− 5.45	9.3E−04	6.0E−02	9.0E+04	4.2E+05	− 5.22
Otu0027/Streptococcaceae	5.0E−04	3.5E−02	8.2E+04	4.1E+04	4.31	3.5E−03	7.5E−02	7.8E+04	4.0E+04	4.28					
Otu0191/Rhodocyclaceae—C39						2.4E−04	2.4E−02	3.3E+03	8.2E+01	3.2	2.1E−02	2.2E−01	2.5E+03	0.0E+00	3.09
Otu0180/RF39_genus	1.3E−02	1.6E−01	8.9E+01	1.7E+03	− 2.91						1.3E−04	2.8E−02	5.1E+01	2.8E+03	− 3.14

## Discussion

Microbial communities in the gastrointestinal tract comprise at least 100 trillion microorganisms, of which the largest number per unit volume is harbored by the colon. Earlier studies report a potential role for intestinal pathogenic bacteria, including adherent-invasive *Escherichia coli* strains^19^ and *Mycobacterium avium* subspecies *paratuberculosis* (MAP)^20,21^, in development of IBDs; however, a study of subjects undergoing antibiotic therapy for up to 2 years does not support a clear role for infectious agents in the pathogenesis of CD^22^. More recently, however, it was suggested that IBD may underly multiple intestinal immunopathologic processes resulting from an interaction between IBD genetic load and gut dysbiosis^23^. Thus, dysbiosis may play a major role in the pathogenesis of IBDs, mainly CD^23,24^. Animal models of intestinal inflammation suggest that dysbiosis initiated by acute pathogenic infection affects gut immunity in a manner that promotes chronic gut inflammation^25^. Gut dysbiosis is associated with decreased abundance of *Firmicutes* and *Bacteroidetes*^26^, including several *Clostridia* (e.g., *Oscillospira*^27^ and *Faecalibacterium prausnitzii*^28^), and with increased abundance of *Enterobacteriaceae*^29^, *Pasteurellaceae*, and *Veillonellaceae*.

Noninvasive fecal sampling has been used to examine the composition of the normal gut microbiome and to investigate gut dysbiosis; however, our knowledge of the gastric microbiota, particularly that in gastric fluid, is rather limited. Here, we examined CD-related dysbiosis of the gastric mucosa and the gastric fluid by 16S rRNA gene amplicon sequencing. The final results of our metagenomic analyses revealed that only one of 24 CD patients was HP-positive, compared with 6 of 19 control subjects. The lower prevalence of HP infection in CD patients may be due to frequent use of antibiotics and immunosuppressants, or to as-yet-unknown protective immune and infectious mechanisms^30^.

Depending on the population, 5–70% of all IBD cases demonstrate non-specific involvement of the stomach (this is particularly true for CD); in addition, 30–80% of CD patients show either endoscopic or histologic changes^3^.

Twenty one of 24 (87.5%) our CD patients revealed different endoscopic abnormalities in the upper GIT; redness and edema of gastric mucosa, BJA and antral erosions were the most frequently observed lesions. Duodenal ulcer was diagnosed in three CD patients; one of them had also irregular erosions and multiple ulcers located at the antrum and pylorus. All patients presented chronic HP-negative gastritis, which was mostly inactive and, usually, mild. None of the patients had gastric mucosal granulomas. In contrast, normal gastric mucosa was found in 9 of 13 (69%) HP-negative control subjects.

The most common cause of gastritis is HP infection; HP-negative gastritis is relatively infrequent, representing only 1.5–21% of all cases^31,32^. By contrast, 70% of CD patients show evidence of microscopic inflammation, presenting as HP-negative gastritis in 10–60% of patients^3,33^. According to the Kyoto global consensus, HP-negative gastritis may be caused by other bacteria, including *H. heilmannii*, *Enterococcus*, *Mycobacteria*, and *Syphilis*^34^. Gantuya et al. examined the gastric microbiota in Mongolian patients with HP-negative and HP-positive gastritis; they concluded that *Streptococcus *sp., *Hemaemophilus parainfluenzae*, and *Treponema *sp. are candidate bacterial species underlying HP-negative gastritis^32^.

It is unclear whether HP-negative diffuse chronic gastritis is clinically significant for those with CD, or whether it is a symptom of an activated immune system^31^. In HP-negative individuals, *Firmicutes*, *Bacteroidota*, and *Actinobacteria* are the most abundant phyla in the gastric mucosa; the most common bacterial genera are *Streptococcus* (phylum *Firmicutes*), *Prevotella* and *Porphyromonas* (*Bacteroidota*), and *Neisseria* and *Haemophilus* (*Proteobacteria*)^6^. We found that *Bacteroidota* and *Firmicutes* were the predominant phyla, accounting for ~ 70% of a total bacteria. *Proteobacteria* was the third most common phylum, accounting for 20% of identified phyla in both the gastric mucosa and gastric juice from HP-negative control individuals. *Actinobacteriota* and *Fusobacteriota* accounted for ~ 10%, whereas other phyla constituted a negligible percentage. Thus, our results support the overall microbiota composition in the mucosa and gastric juice reported previously^6^.

In accordance with a previous study^6^, in our infected subjects HP accounted for 20–98% of all identified gastric mucosal bacteria and only for 1–13% of the gastric fluid microbiota.

Expectedly, the microbiota in both the mucosa and gastric liquid differed between CD patients and controls. Although changes in the mean abundance of five phyla were similar at all three gastric sites (Table 2), the relative abundance of *Bacteroidota*, *Proteobacteria*, *Fusobacteriota*, and *Campilobacterota* differed significantly in CD patients and control subjects only in gastric corpus samples, whereas *Actinobacteriota* differed significantly only in gastric liquid samples. However, the lack of significance in observed differences at other locations may be explained by the small size of the study groups and high variability in the frequency of individual phyla.

The *Firmicutes/Bacteroidota* ratio in gastric mucosal samples was higher in CD patients than in control subjects; this was not the case for gastric liquid samples. While microbial alpha diversity did not differ between CD patients and controls at any of the tested sites, the beta diversity clustering patterns were different for all three sites.

In at least in one site, the relative abundance of 67 OTUs was significantly different in HP-negative CD patients and control subjects. Of these, four OTUs [Otu0015 (*Neisseriaceae*); Otu0008 (*Neisseria*); Otu0092 (*Absconditabacteriales*); and Otu0170 (*Microbacteriaceae*, *Candidatus_Aquiluna*)] exhibited significantly lower abundance at all test sites in CD patients than in all test sites in control subjects. Previous research focused primarily on the microbiota in gastric mucosal biopsies; few studies have compared the microbiomes in the gastric mucosa and gastric fluid^35,36^. Unlike our research, these studies reported differences in both microbiota composition and abundance between gastric fluid and gastric mucosa; as a consequence, bacteria from gastric juice cannot reflect the composition of gastric mucosa microbiome^35,36^. In our study, the percentage of different bacteria in gastric mucosa and fluid was very similar, although the majority of the statistically different OTUs were found in gastric fluid. However, further research is needed to establish whether gastric dysbiosis underlies the pathogenesis of HP-negative gastritis in CD patients.

Most previous studies of gastric microbiomes report changes in gastric microbiota related to precancerous states and gastric cancer. In one study, 16S rRNA gene sequencing-based analysis revealed higher prevalence of gastric mucosa *Lactobacillus*, *Streptococcus mitis*, *Streptococcus parasanguinis*, *Prevotella*, and *Veillonella* in gastric cancer patients^37^. In the general population (with low HP prevalence), microbial diversity in normal stomach is higher than that in individuals with non-atrophic or atrophic gastritis, which show an increased abundance of pathogenic organisms^38^. Another study reported *Clostridium*, *Fusobacterium*, and *Lactobacillus* genera as highly abundant in patients with gastric cancer ^39^, whereas another suggested that *Peptostreptococcus stomatis*, *Streptococcus* anginosus, *Parvimonas micra*, *Slackia exigua*, and *Dialister pneumosintes* led to progression from a precancerous to a cancerous state^40^.

## Conclusions

While the small size of the studied groups limit the final conclusions, our studyrevealed gastric dysbiosis in patients with CD. Overall, the results show that dysbiosis of bacteria in gastric fluid does not differ from that of bacteria adhering to the gastric mucosa, suggesting that gastric liquid comprises mucosal-resident bacteria rather than those relocated from the oropharynx or esophagus. We also identified marked differences in the microbial profiles of both the gastric mucosa and liquid in HP-negative CD patients and the HP-negative healthy subjects or control individuals with functional dyspepsia; differences were noted in beta diversity, bacterial phyla, and individual taxa. However, since we have not identified individual bacteria with selective pathogenicity, the clinical relevance of our findings is uncertain. We consider gastric dysbiosis as a possible prerequisite for CD pathogenesis. Therefore, further studies should focus on exploring the relationship between host immunity and microbiota dysbiosis at the level of both upper and lower gastrointestinal tract.

## Supplementary Information

Supplementary Table 1.

Supplementary Table 2.
